# Machine learning, clinical-radiomics approach with HIM for hemorrhagic transformation prediction after thrombectomy and treatment

**DOI:** 10.3389/fneur.2025.1471274

**Published:** 2025-02-27

**Authors:** Sheng Hu, Junyu Liu, Jiayi Hong, Yuting Chen, Ziwen Wang, Jibo Hu, Shiying Gai, Xiaochao Yu, Jingjing Fu

**Affiliations:** ^1^Department of Radiology, The Fourth Affiliated Hospital of School of Medicine, and International School of Medicine, International Institutes of Medicine, Zhejiang University, Yiwu, Zhejiang, China; ^2^Department of Neurology, The Fourth Affiliated Hospital of School of Medicine, and International School of Medicine, International Institutes of Medicine, Zhejiang University, Yiwu, Zhejiang, China; ^3^Department of Neurosurgery, The Fourth Affiliated Hospital of School of Medicine, and International School of Medicine, International Institutes of Medicine, Zhejiang University, Yiwu, Zhejiang, China

**Keywords:** hemorrhagic transformation, machine learning, thrombectomy, acute ischemic stroke, multi-detector CT

## Abstract

**Background:**

This study aimed to develop a clinical-radiomics model using hyperattenuated imaging markers (HIM), characterized by hyperattenuation on head non-contrast computed tomography immediately after thrombectomy, to predict the risk of hemorrhagic transformation (HT) in patients undergoing endovascular mechanical thrombectomy (MT).

**Methods:**

A total of 159 consecutive patients with HIM were screened immediately after MT for inclusion. The datasets were randomly divided into training and test cohorts at a ratio of 8:2. An optimal machine learning (ML) algorithm was used for model development. Subsequently, models for clinical, radiomics, and clinical-radiomics were developed. The performance of the models was measured using receiver operating characteristic (ROC) and decision curve analyses (DCA). The interpretability and predictor importance of the model were analyzed using Shapley additive explanations.

**Results:**

Of the 159 patients, 100 (62.9%) exhibited HT. The support vector machine (SVM) was the optimal ML algorithm for constructing the models. In predicting HT, the areas under the curve (AUCs) of the clinical model were 0.918 (95% confidence interval [CI] = 0.869–0.966) in the training cohort and 0.854 (95% CI = 0.724–0.984) in the test cohort. The AUCs of the radiomics model were 0.869 (95% CI = 0.802–0.936) and 0.829 (95% CI = 0.668–0.990), while those of the clinical-radiomics model were 0.944 (95% CI = 0.905–0.984) and 0.925 (95% CI = 0.832–1.000).

**Conclusion:**

The suggested clinical-radiomics model based on HIM is a reliable method that can provide a risk evaluation of HT in individuals undergoing MT.

## 1 Introduction

Endovascular mechanical thrombectomy (MT) is the recommended treatment for acute ischemic stroke (AIS) because it improves reperfusion rates and clinical outcomes (1). Despite advancements in treatment strategies, hemorrhagic transformation (HT) remains a significant determinant of patient prognosis, with over 50% of patients experiencing unfavorable outcomes (2). Symptomatic intracerebral hemorrhage can lead to death in some patients (3). Furthermore, asymptomatic intracerebral hemorrhage and subarachnoid hemorrhage (SAH) contribute to adverse outcomes (4, 5). Appropriate postoperative treatment can improve the prognosis. Therefore, early prediction of HT is crucial for adjusting treatment after intervention. It is particularly important to determine whether blood pressure control and drug therapy should be initiated to prevent early re-occlusion of blood vessels caused by endothelial injury.

The hyperattenuated imaging marker (HIM) detected immediately on non-contrast computed tomography (NCCT) after MT is the most accessible and earliest imaging marker for predicting postoperative complications (6, 7). HIM represents areas of hyperattenuation on post-thrombectomy cranial NCCT scans, often including iodine contrast extravasation (ICE) and occasionally containing hemorrhage. Postoperatively, these extravasated contrast agents are gradually absorbed, while hemorrhage may either start or increase. Numerous studies have reported the postoperative presence of HIM in patients, ranging from 32.9 to 87.5% (8, 9). The presence of HIM indicates disruption of the blood–brain barrier and blood-cerebrospinal fluid barrier, which are closely related to bleeding, making it the most commonly used imaging marker for hemorrhage prediction after surgery (4, 7, 10, 11). Several studies have demonstrated that the presence of metallic hyperdensity signs and cortical involvement in HIM are more sensitive and specific for predicting HT (12, 13). However, there is currently no standardized predictive method with acceptable sensitivity and specificity that can accurately predict HT in all patients with HIM.

Radiomics, a technique that involves the extraction of numerous characteristics from medical images, is becoming increasingly popular for improving the diagnosis and management of ischemic stroke. However, previous studies using radiomic features based on HIM have primarily focused on detecting hemorrhage in the brain parenchyma, overlooking the inclusion of SAH (14, 15). In reality, both intracerebral hemorrhage and SAH can affect the subsequent use of anticoagulants. Accordingly, it is important to consider both types of hemorrhage when using radiomics for the prediction and treatment planning of patients with ischemic stroke.

Machine learning (ML) can be used to integrate radiomic features with high-risk clinical factors to develop a hemorrhage prediction model for stroke (16, 17). Consequently, this study aimed to use radiomic attributes derived from HIM in NCCT and clinical characteristics to develop a predictive model for both intracerebral hemorrhage and SAH in patients undergoing MT using ML algorithms.

## 2 Materials and methods

### 2.1 Ethical approval of the study protocol

Approval Number KY2023112 was granted by the Ethics Committee of the Fourth Affiliated Hospital, Zhejiang University College of Medicine. The committee exempted informed consent. All clinical studies followed the principles of the Declaration of Helsinki.

### 2.2 Patients and study design

A retrospective review was conducted on patients who experienced AIS due to blockage in the large blood vessels inside the skull and received endovascular MT between June 2016 and November 2021 at the Fourth Affiliated Hospital, Zhejiang University School of Medicine. The latest guidelines were followed at the time, and they included the indications and contraindications of MT and thrombolysis. The general clinical features, laboratory examinations, clinical presentations, and imaging data of the patients were collected within the past 90 days.

**Figure 1:**
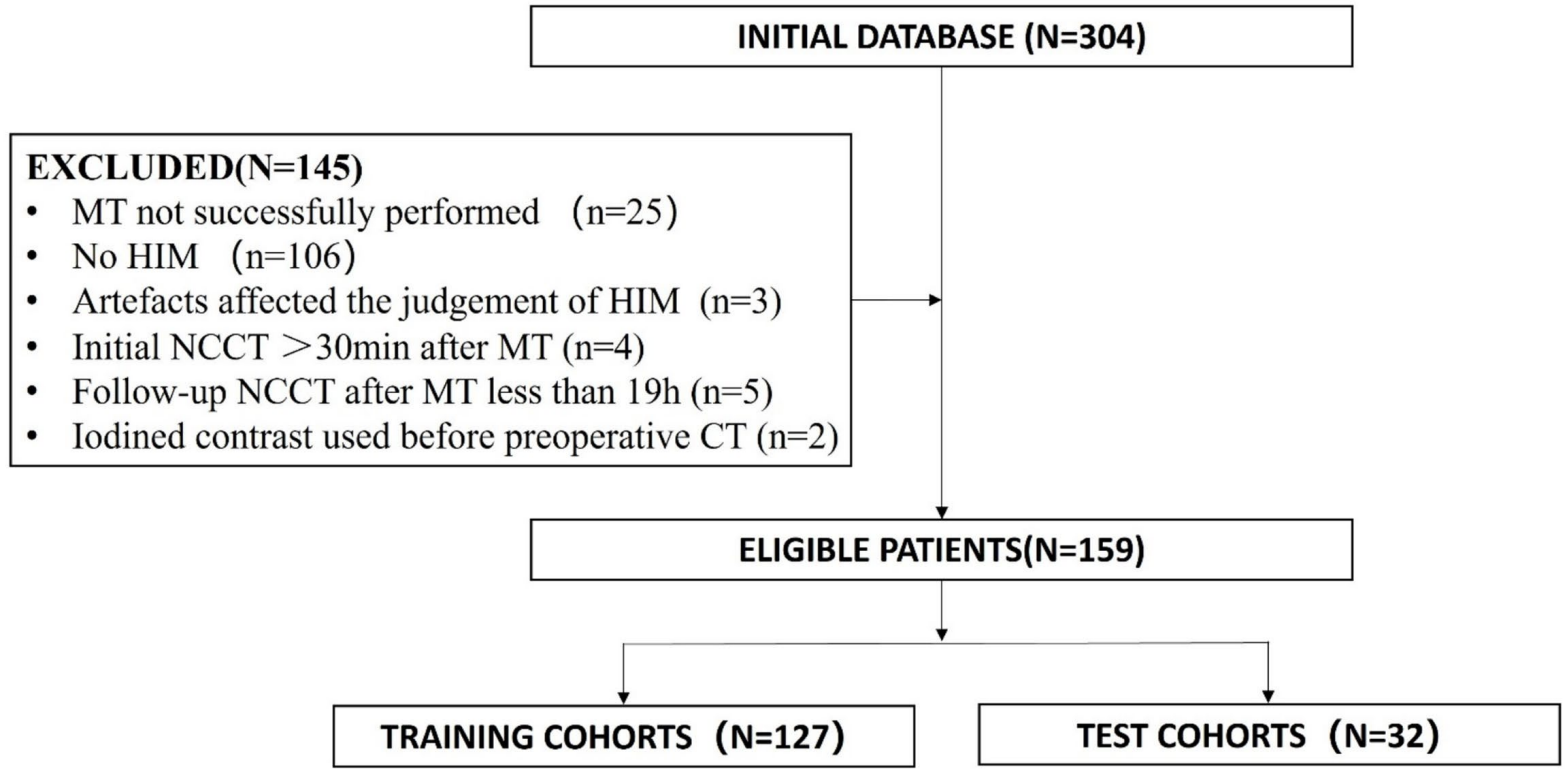
The flowchart of the patient recruitment pathway. MT, mechanical thrombectomy; NCCT, non-contrast CT; HIM, Hyperattenuated Imaging Marker.

The inclusion criteria were as follows: (1) Patients scheduled for MT. (2) Patients who underwent head NCCT or magnetic resonance imaging (MRI) after MT; (3) initial postoperative head NCCT was conducted within 30 min after MT; (4) HIM, which is defined as an area of hyperattenuation visible in the brain parenchyma and subarachnoid space, was detected on the initial head NCCT following MT. The exclusion criteria included the following: (1) Patients for whom MT was aborted due to unfavorable vascular anatomy or who were switched to medical therapy; (2) NCCT follow-up time after MT of less than 19 h (18); (3) the judgment of HIM or hemorrhage was influenced by artifacts like metal or motion artifacts; (4) the use of iodinated contrast before preoperative CT affected the determination of HT. Figure 1 displays a flowchart illustrating the study participants.

### 2.3 Imaging

Within 30 min after thrombectomy, initial postoperative head NCCT was performed using a 64-row spiral CT scanner (Somatom Definition AS, Siemens Healthineers, Forchheim, Germany) and a 62-row spiral scanner (Optima CT620, GE Medical Systems, Milwaukee, WI, US). The scanning parameters included an axial mode with a tube voltage of 120 kVp and a tube current of 250–300 mAs. The scanning range was extended from the skull base to the cranial roof with a section thickness of 5 mm. Reconstruction was performed using the standard algorithm. Two neuroradiologists with over a decade of professional experience evaluated the imaging data randomly and were unaware of the clinical circumstances. Two evaluators discussed conflicting images to reach a consensus.

### 2.4 Reference standard

The HT was classified according to the Heidelberg Classification, adhering to the following criteria: 1. Hyperattenuation remained evident on the 24-h CT scan, and 2. Follow-up CT or MRI conducted within 90 days after surgery revealed hemorrhage within the range of infarction. The maximum Hounsfield unit (HUmax) was defined as the highest HU value within a specified Region of Interest (ROI), measuring 3 × 3 pixels. Two neuroradiologists (S.H. and Z.W.) with >5 years of experience independently evaluated the training and test groups. They were blinded to patient outcomes and assessed the groups separately. Any discrepancy was resolved by consensus.

### 2.5 Data preprocessing

This study included 159 patients. Patients were randomly divided into a training cohort (127 patients) and a test cohort (32 patients). Data on several baseline and preprocedural factors were collected, including age, gender, diabetes mellitus, coronary artery disease, atrial fibrillation, hypertension, smoking and drinking, thrombolysis, and baseline National Institute of Health Stroke Scale (NIHSS) score. These data were obtained by reviewing the patient’s medical records, procedure notes, and progress and follow-up notes. Two experienced neuroradiologists, who were unaware of the patient’s clinical information, determined the baseline Alberta Stroke Program Early CT Score (ASPECTS) in the anterior and posterior circulation, subarachnoid HIM (sHIM), and HUmax through discussion. Furthermore, data regarding the surgical procedure, including the type of stent, number of stent passes, stent placement, and modified Thrombolysis in Cerebral Infarction scores, were collected.

### 2.6 Radiomics features extraction

On each initial postoperative CT image, the ROI of the HIM was manually segmented along the HIM contour, incorporating both parenchyma and sulcus. This process was conducted using the ITK-SNAP software (version 3.8.0^1^). Both radiologists were blinded to the clinical information and final outcomes.

To eliminate any potential variations in CT images obtained using different CT scanners, NCCT images were reconstructed using a voxel size of 1 × 1 × 1 mm^3^ and gray-scale discretization. Two radiologists independently segmented the images of every HIM and measured them using a method that ensured double anonymization. An intraclass correlation coefficient (ICC) ≥ 0.75 was considered robust.

A Pyradiomics in-house feature analysis program^2^ was used to extract radiomic features from HIM in NCCT. From the NCCT images, 1834 radiomics features were extracted. Various techniques were used to extract texture features, including first-order statistics, shape-based analysis, gray-level co-occurrence matrix (GLCM), gray-level run length matrix (GLRLM), neighboring gray-tone difference matrix (NGTDM), gray-level size zone matrix (GLSZM), and gray-level dependence matrix (GLDM).

### 2.7 Finding the best ML algorithm to build models

All clinical data and robust radiomics features were combined to develop the combined models. A Mann–Whitney *U* test and feature screening were performed for all features. The features with a *p*-value <0.05 were included. Spearman’s rank correlation coefficient was calculated for the features exhibiting high repeatability to evaluate the association between features. If the correlation coefficient between any two characteristics >0.9, one of the characteristics would be preserved. To ensure a comprehensive depiction of the features, a greedy recursive deletion strategy was used for feature filtering. This process includes removing the characteristics with the highest duplication in the existing collection during each cycle. For signature construction, the discovery dataset was subjected to the least absolute shrinkage and selection operator (LASSO) regression model. By applying a regularization weight *λ*, LASSO reduces the regression coefficients to zero and assigns a value of zero to the coefficients of the irrelevant features. To determine the best *λ*, a 10-fold cross-validation was applied, and the *λ* value that resulted in the lowest cross-validation error was selected using the minimum criteria. The included features with coefficients that were not zero were used to fit the regression model and were incorporated into the model. LASSO regression modeling was performed using the Python scikit-learn library.

Following the LASSO feature screening, the selected features were incorporated into the ML models. To develop the HT prediction model, the effects of seven ML algorithms [Support Vector Machine (SVM), Light Gradient Boosting Machine, k-nearest neighbor, random forest, AdaBoost, eXtreme Gradient Boosting, and Gradient Boosting] were compared before modeling. The optimal algorithm was identified through 5-fold cross-validation in the training cohort. The SVM model for ML was identified as having the greatest mean area under the curve (AUC) of the receiver operating characteristic (ROC) curve in the test group (Table 1). Hence, the chosen characteristics were fed into the SVM algorithms to develop the risk model. To obtain the optimized subset of features, a cross-verification was conducted using a 5-fold method.

**Table 1:** Performance of ML combined models.

Model	Cohort	Accuracy	AUC	95% CI	Sensitivity	Specificity	PPV	NPV	Precision	Recall	F1
SVM	Train	0.89	0.944	0.9046–0.9837	0.938	0.809	0.893	0.884	0.893	0.938	0.915
SVM	Test	0.875	0.925	0.8325–1.0000	0.9	0.833	0.9	0.833	0.9	0.9	0.9
KNN	Train	0.858	0.912	0.8626–0.9619	0.863	0.851	0.908	0.784	0.908	0.863	0.885
KNN	Test	0.844	0.842	0.6900–0.9934	0.95	0.727	0.826	0.889	0.826	0.95	0.884
Random-Forest	Train	1	1	1.0000–1.0000	1	1	1	1	1	1	1
Random-Forest	Test	0.719	0.815	0.6669–0.9623	0.55	1	1	0.571	1	0.55	0.71
XGBoost	Train	1	1	1.0000–1.0000	1	1	1	1	1	1	1
XGBoost	Test	0.75	0.717	0.5157–0.9176	0.8	0.667	0.8	0.667	0.8	0.8	0.8
LightGBM	Train	0.843	0.921	0.8765–0.9647	0.875	0.787	0.875	0.787	0.875	0.875	0.875
LightGBM	Test	0.844	0.846	0.7015–0.9902	0.9	0.75	0.857	0.818	0.857	0.9	0.878
Gradient-Boosting	Train	0.953	0.994	0.9873–1.0000	0.925	1	1	0.887	1	0.925	0.961
Gradient-Boosting	Test	0.75	0.708	0.5033–0.9133	0.85	0.778	0.773	0.7	0.773	0.85	0.81
AdaBoost	Train	0.85	0.947	0.9132–0.9799	0.775	0.979	0.984	0.719	0.984	0.775	0.867
AdaBoost	Test	0.75	0.815	0.6683–0.9609	0.75	0.75	0.833	0.643	0.833	0.75	0.789

### 2.8 Building models

**Figure 2:**
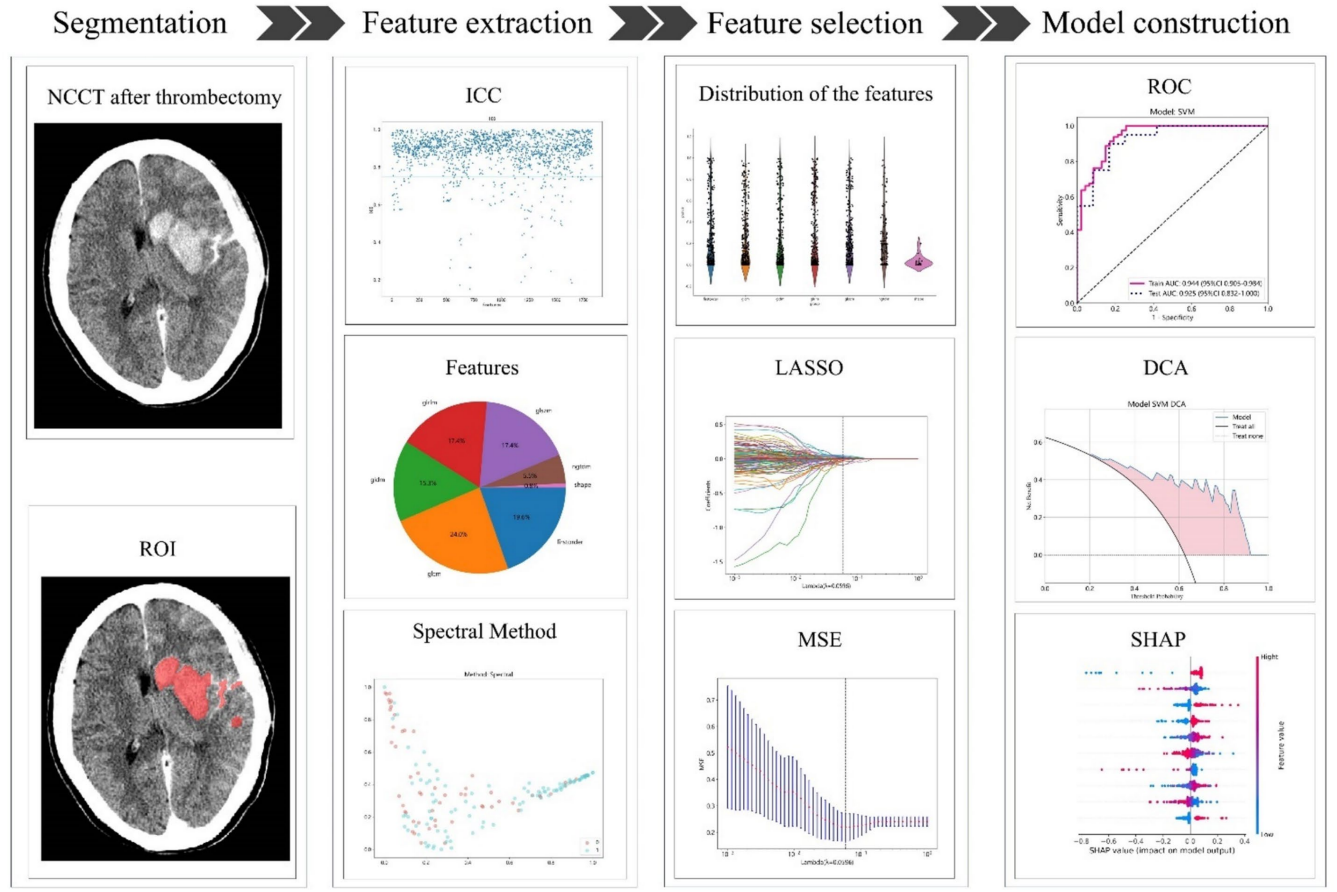
The process of building the radiomics model. NCCT, non-contrast CT; ROI, region of interest; ICC, intraclass correlation coefficient; LASSO, least absolute shrinkage and selection operator; MSE, mean square error; ROC, receiver operating characteristic; DCA, decision curve analysis; SHAP, Shapley Additive explanations.

A radiomics model was developed for the training group using the most important radiomics characteristics that were ultimately chosen following the same procedure as that used for the combined model. Additionally, the radiomics model was validated in the test cohort. In the training cohort, the collected clinical characteristics were integrated into the LASSO regression model to identify the most significant characteristics, retaining only those with coefficients that were not zero. The SVM algorithm was then used to develop a clinical model by employing these characteristics. The efficiency of the model was evaluated using the test cohort. A flowchart of the radiomics model construction is shown in Figure 2.

The combined model was developed in the training cohort and was subsequently verified in the test cohort. The diagnostic efficacy of the combined model was evaluated using ROC curves. To assess the clinical applicability of the predictive models, a decision curve analysis (DCA) was conducted. Furthermore, the performance of these models was evaluated using Shapley Additive explanations (SHAP) analysis to determine the significance of each feature in predicting HT.

### 2.9 Statistics

The statistical package for the social sciences software (version 26.0, IBM) was used to perform statistical analyses. For continuous variables, the disparity was determined using either the Student’s *t*-test or the Mann–Whitney *U* test, depending on suitability. Continuous data are presented as mean ± standard deviation or median with interquartile range, depending on the distribution of the data. Differences in categorical variables were determined using either the Chi-square or Fisher’s exact test, depending on the circumstances. The significance threshold for all statistical tests was set at *p* < 0.05. Python software (version 3.11.1^3^) was used to perform feature extraction and screening and to construct the models. The performance of the three models was evaluated using the AUC, 95% CI, accuracy, sensitivity, and specificity.

## 3 Results

### 3.1 Comparison of patient clinical characteristics

This study included 159 patients diagnosed with HIM, with 127 and 32 patients assigned to training and test cohorts, respectively. The initial attributes of patients in both the training and test groups are listed in Table 2. HT occurred in 100 of the 159 patients. The training and test cohorts differed significantly in thrombolysis (*p* = 0.027).

**Table 2:** Characteristics of the training and test sets.

Feature name	Train	Test	*P*-value
Age, mean ± SD	67.22 ± 13.84	66.44 ± 15.59	0.781
Men, *n*(%)	81(63.78)	20(62.50)	0.894
Atrial fibrillation, *n*(%)	48(37.80)	9(28.13)	0.311
Hypertension, *n*(%)	72(56.69)	20(62.50)	0.555
Diabetes mellitus, *n*(%)	17(13.39)	4(12.50)	0.896
Coronary artery disease, *n*(%)	20(15.75)	8(25.00)	0.060
Drinking, *n*(%)	27(21.26)	8(25.00)	0.651
Smoking, *n*(%)	29(22.83)	9(28.13)	0.534
Thrombolysis, *n*(%)	48(37.80)	19(59.38)	0.027*
Baseline NIHSS, median (Q1, Q3)	16(12–20)	15(13–18)	0.893
Anterior circulation, *n*(%)	119(93.70)	30(93.75)	0.992
ASPECTS, median (Q1, Q3)	9(8–10)	9(8–10)	0.504
Humax ≥ 90, *n*(%)	35(25.55)	14(43.75)	0.077
sHIM, *n*(%)	46(36.22)	13(40.62)	0.647
mTICI 2b or 3, *n*(%)	113(88.98)	26(81.25)	0.242
Stent type, *n*(%)			0.486
SOLITAR	71(55.91)	19(59.38)	
TREVO	25(19.69)	5(15,63)	
SOLITAR + TREVO	16(12.60)	4(12.50)	
others	15(11.81)	4(12.50)	
Pass number, median (Q1, Q3)	2(1–3)	2(1–3)	0.958
Stent implantation, *n*(%)	25(19.69)	8(21.88)	0.784

*Represents *P* < 0.05; SD, standard deviation; HU, Hounsfield; NIHSS, National Institutes of Health Stroke Scale; Q1, first quartile; Q3, third quartile; ASPECTS, Alberta Stroke Program Early CT Score; mTICI, modified thrombolysis in cerebral infarction.

### 3.2 Establishment and performance of the radiomics model

A total of 1834 radiomic characteristics were obtained from the NCCT images after MT, with 1,681 characteristics (ICC ≥ 0.75) demonstrating acceptable consistency between observers. To develop the model, five features per patient were selected by excluding feature pairs with strong correlations.

**Figure 3:**
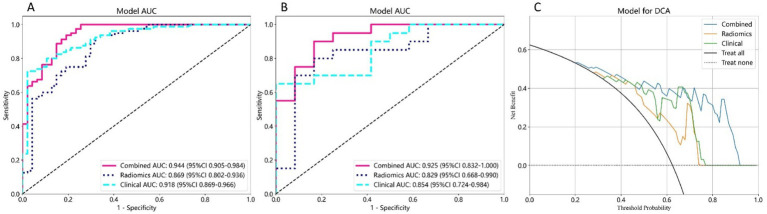
Receiver operating characteristic (ROC) and DCA curves of the three models. **(A)** ROC curves for predicting HT in the training cohort. **(B)** The corresponding ROC curves for the test cohort. **(C)** DCA curves for the three models. The *y*-axis represents the net benefit, and the *x*-axis represents the threshold probability. The blue line, green line, and orange line represent the expected net benefit of predicting HT using the combined model, clinical model, and radiomics model, respectively. ROC, receiver operating characteristic; DCA, decision curve analysis; HT, hemorrhagic transformation.

In the training group, the radiomics model exhibited an AUC of 0.869 (95% confidence interval [CI] = 0.802–0.936) with sensitivity and specificity rates of 0.925 and 0.681, respectively. The test group exhibited an AUC of 0.829 (95% CI = 0.668–0.990) and well-balanced sensitivity (0.800) and specificity (0.833) (Table 3 and Figure 3).

**Table 3:** Performance of the three models.

Model	Cohort	Accuracy	AUC	95% CI	Sensitivity	Specificity	PPV	NPV	Precision	Recall	F1
Combined	Train	0.890	0.944	0.9046–0.9837	0.938	0.809	0.893	0.884	0.893	0.938	0.915
Combined	Test	0.875	0.925	0.8325–1.0000	0.9	0.833	0.9	0.833	0.9	0.9	0.9
Radiomics	Train	0.835	0.869	0.8018–0.9365	0.925	0.681	0.831	0.842	0.831	0.925	0.876
Radiomics	Test	0.812	0.829	0.6679–0.9905	0.8	0.833	0.889	0.714	0.889	0.8	0.842
Clinical	Train	0.819	0.918	0.8686–0.9665	0.725	0.979	0.983	0.676	0.983	0.725	0.835
Clinical	Test	0.781	0.854	0.7242–0.9841	0.65	1	1	0.632	1	0.65	0.788

### 3.3 Performances of the clinical models

Nine clinical factors, including HUmax ≥90, anterior circulation, sHIM, ASPECTS, male sex, drinking, coronary artery disease, baseline NIHSS score, and pass number, were selected after implementing LASSO feature screening. These factors were used to develop the clinical model.

In the training cohort, the clinical model demonstrated an AUC of 0.918 (95% CI = 0.869–0.966), a sensitivity of 0.725, and a specificity of 0.979. Similarly, the clinical model exhibited an AUC of 0.854 (95% CI = 0.724–0.984) in the test cohort with a sensitivity of 0.650 and specificity of 1.0. These results are presented in Table 3 and Figure 3.

### 3.4 Performances of the combined model

The models were developed by combining all the clinical data and robust radiomics features. After excluding the feature pairs with strong correlations, 17 features per patient were selected for further analysis. These results are presented in Table 3 and Figure 3.

In the training cohort, the combined model exhibited an AUC of 0.944 (95% CI = 0.905–0.984), with sensitivity and specificity rates of 0.938 and 0.809, respectively; whereas the AUC was 0.925 (95% CI = 0.832–1.000) in the test cohort, and the sensitivity and specificity were 0.900, and 0.833, respectively (Table 3 and Figure 3). The Delong test showed no significant differences between models: combined vs. radiomics (*p* = 0.172), combined vs. clinical (*p* = 0.316), and radiomics vs. clinical (*p* = 0.800). The DCA values of the three models for both the training and test cohorts are illustrated in Figure 3C. The DCA demonstrated that the combined model provided a significant clinical prediction advantage for the majority of threshold probabilities compared to the clinical and radiomics models.

### 3.5 Explanation and visualization of the combined model

**Figure 4:**
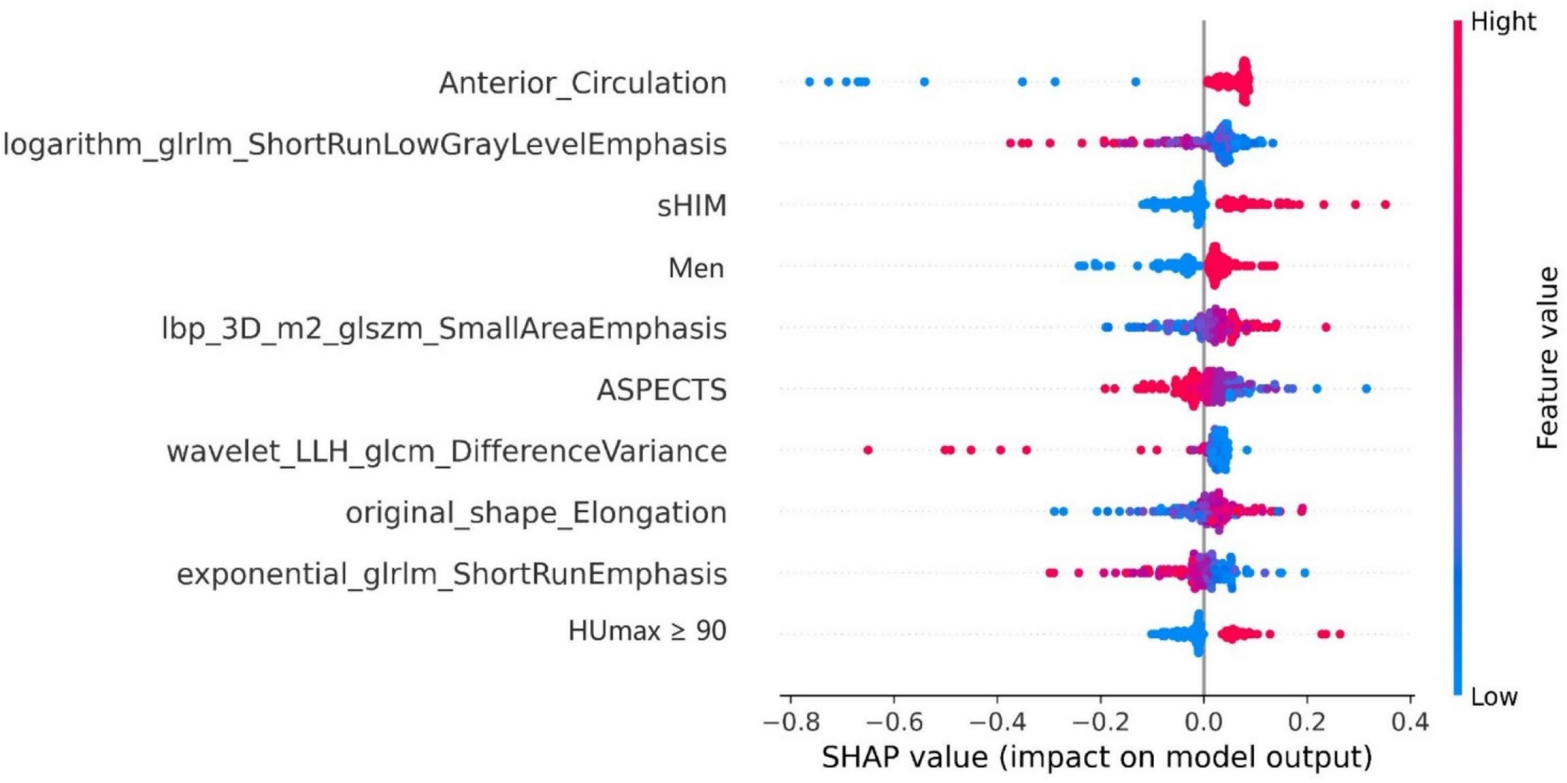
The rank of importance for each feature in the combined model for HT prediction. The SHAP summary plot illustrates the distribution of patient features, arranged by importance, with the most critical feature at the top and the least important at the bottom. The color legend on the right side represents the influence of feature values on the outcome, with red indicating high values and blue indicating low values. Positive SHAp values (right on the *x*-axis) suggest a higher probability of HT, while negative values (left on the *x*-axis) indicate the opposite. SHAP, Shapley Additive exPlanations; sHIM, subarachnoid hyperattenuated imaging markers; ASPECTS, Alberta Stroke Program Early CT Score.

After performing the prediction modeling of HT, the feature importance matrix plot was used to rank the most important variables. The plot revealed the influence of each factor on HT prediction. To demonstrate the influence of each characteristic on the model’s prediction, a summary chart of the SHAP values is depicted in Figure 4, illustrating the correlation between high or low SHAP values and the prediction model. The blue dots, which indicate lower values of logarithm_glrlm_ShortRunLowGrayLevelEmphasis, tend to be more concentrated in areas with a higher likelihood of HT. These results indicate that the SHAP values of this measurement exhibited an inverse relationship with the risk of HT occurrence. Contrarily, the risk of HT was high on the side, with a higher frequency of red dots indicating high Anterior_Circulation, Ibp_3D_m2_glszm_SmallAreaEmphasis, sHIM, and men, implying a positive correlation between the SHAP value of this index and the risk of HT.

## 4 Discussion

Hemorrhagic transformation is a significant complication of MT in patients with AIS. Identifying patients at risk of developing HT is crucial for optimizing the management of stroke patients. We aimed to address this requirement by developing and evaluating interpretable ML models. The occurrence of HT after MT was predicted using baseline clinical information and imaging features based on HIM in these models. Our combined model, incorporating both clinical factors and radiomics features, demonstrated superior predictive performance. This model yielded an AUC of 0.944 in the training group and 0.925 in the test group. These results highlight the potential of our model to accurately predict the risk of post-MT HT in patients with AIS. The predictive capacity of the model was improved by combining clinical and radiomics characteristics, providing valuable information to optimize patient care and minimize the incidence of this severe complication.

The occurrence of HT is common after MT, with the most common type being intracerebral hemorrhage. Recent studies have demonstrated an increase in the number of reports of SAH as the indications for MT have expanded. In a study of anterior circulation MT, the incidence of SAH was 13.2%, and the incidence of intracerebral hemorrhage was 39.6%. SAH accounted for 1/4 of all hemorrhages (19). Intracerebral hemorrhage and SAH after thrombectomy mutually influence each other. Disruption of the blood–brain barrier or injury to the vascular wall in the brain parenchyma leads to SAH (4, 20), and vascular wall injury can also cause parenchymal damage (21, 22). Patients who develop SAH after thrombectomy have a poor prognosis, which is further exacerbated by the presence of intracerebral hemorrhage (23–25). SAH, like intracerebral hemorrhage, also affects timely postoperative treatment. However, multiple studies have separated them for analysis and have not included SAH as an outcome measure in predictive models (12, 26, 27). Optimal management and treatment strategies for patients with AIS can be achieved through the precise and prompt prediction of hemorrhage after thrombectomy. Patients without hemorrhage should receive appropriate perfusion maintenance and prevention of vessel re-occlusion to salvage more brain tissue. Patients with hemorrhage should have their blood pressure lowered and antiplatelet aggregation drugs discontinued to prevent further bleeding. This study included both intracerebral hemorrhage and SAH as outcome measures and achieved satisfactory predictive results.

HIM-based radiomics has demonstrated the HT prediction potential. Chen et al. (14) conducted a study using radiomic features of HIM within 1 h after MT to determine the presence of hemorrhage, achieving an AUC of 0.826. Ma et al. (15) developed a model that combined radiomics features of intracerebral HIM within 24 h post-surgery with clinical characteristics to predict HT. Their model demonstrated an AUC of 0.926 for intracerebral hemorrhage occurring 48 h after surgery. However, they used a broad definition of HIM and hemorrhage timing, which may have inadvertently excluded certain instances of HIM and hemorrhage that have been resolved.

Contrarily, our study followed stricter inclusion criteria that restricted the examination time of HIM to a maximum of 30 min. But the strict 30-min post-MT imaging window may limit generalizability. Future studies should broaden this timeframe to minimize potential bias. Moreover, unlike their study, we included SAH as an outcome measure, which is more applicable in clinical practice. Our clinical radiomics model demonstrated the best predictive performance, with AUCs of 0.944 and 0.925 in the training and test cohorts, respectively, confirming its superior predictive capability further, even though the Delong test did not reveal statistically significant differences between models.

Support vector machine, a widely utilized ML algorithm, is frequently used for tasks involving recognition and classification. The SVM has become a prominent algorithm for supervised learning and is widely used in the fields of pattern recognition and ML. The algorithm uses a linear decision boundary known as a hyperplane to identify the optimal hyperplane that maximizes the gap distance between two categories (28). Furthermore, SVM can address non-linear classification problems by transforming data into a space with more dimensions through diverse kernel functions. In previous studies, SVM has been successfully applied in neuroimaging to predict SAH and prognosis after treatment (16, 29). This study involved the development of seven different predictive models for HT, with the SVM model exhibiting superior performance. Moreover, we used SHAP, a game-theory-based method, to visually represent the impact of individual features in the combined model for prognostic forecasting. Our SHAP analysis identified several radiomic features with clinical relevance (30). Among the positively correlated features, elevated SmallAreaEmphasis values suggest densely clustered small-sized hyperdense foci within HIM regions. These micro-foci likely represent early microhemorrhages or ICE, and their aggregation indicates exacerbated blood–brain barrier disruption, which may predispose patients to HT following reperfusion therapy. Elongation exhibited a positive correlation with hemorrhage risk. HIM with an elongated shape predominantly localized to cortical regions may reflect the high metabolic demand of cortical tissue, which can amplify ischemia–reperfusion injury (8). Conversely, negatively correlated features revealed protective imaging patterns. Reduced DifferenceVariance signifies a homogenized grayscale distribution within HIM, suggesting potential alignment with infarct core regions characterized by severe blood–brain barrier damage, thereby elevating hemorrhage susceptibility (31). The negative correlations observed for ShortRunLowGrayLevelEmphasis and ShortRunEmphasis indicate diminished low-density areas and a lack of short-run texture structures within HIM. This further aligns with studies suggesting that lesions dominated by homogeneous hyperdensity are more prone to hemorrhagic complications (12).

The top nine predictive indicators in the combined model included clinical factors such as anterior circulation, sHIM, male gender, ASPECTS score, and HUmax ≥90. Morhard et al. (32) conducted a study comparing the incidence of HIM in patients with anterior and posterior circulation stroke using dual-energy CT. The results revealed that the incidence of HIM was significantly higher in patients with anterior circulation stroke than in those with posterior circulation stroke, leading to an increased rate of HT. This difference can be attributed to the relatively small volume of the posterior circulation area and limited resolution of the posterior fossa CT caused by beam-hardening artifacts. Manual ROI delineation may miss subtle HIM regions. Future work should prioritize automated segmentation tools to improve consistency. Kim et al. (33) discovered that the infarcted core was larger in the sHIM group, indicating a poor collateral circulation status, thereby leading to the expansion of the progressive infarcted core and an increased risk of HT (34). Besides, sHIM could result from arterial injury caused by the procedure, indicating the presence of significant intracranial hemorrhage. This finding aligns with those of the present study. The risk of HT after MT was comparable between men and women. However, in Asian populations, there is a stronger association between male gender and HT (35–37). Further evidence is required to confirm these observations. Numerous studies have demonstrated that a low preoperative ASPECTS may indicate severe damage to the blood–brain barrier due to ischemia (7, 38–41). This damage increases the risk of HT after MT. Additionally, the maximum CT value, especially when the threshold is set at 90 HU, indicates impairment of the blood–brain barrier and acts as a reliable indicator of the risk of HT (7, 8, 12, 42).

Our study has several limitations that should be addressed. First, the retrospective nature of the study, its single-center design, and its relatively small sample size may have introduced selection bias and overestimation of our findings. We are actively collaborating with external institutions to conduct prospective multicenter validation. Additionally, we plan to implement local prospective internal validation in ongoing studies. Until such external corroboration is achieved, clinical application of the model requires cautious interpretation, and its generalizability remains to be further verified. Second, the manual segmentation process for lesions in our study was time-consuming and complex, especially for lesions with indistinct boundaries. Consequently, future research endeavors should prioritize the improvement of automatic segmentation technology to achieve a satisfactory level of reliability and reproducibility. Third, the small sample size and the imbalance between the two groups in our study may have affected the statistical power and generalizability of the results. Larger sample sizes and improved balance between the groups should be considered in future studies. Fourth, our approach relies on conventional radiomics characteristics. The prediction ability of the model can be improved by incorporating a deep-learning approach. Finally, interpreting the radiomics features used in our study presents a formidable challenge to understanding their fundamental biological significance. To improve our understanding of their biological significance, future research should focus on investigating the association between the histological attributes of thrombi and radiomics features.

## 5 Conclusion

This study involved the development of a model that integrates radiomics characteristics obtained from NCCT images of HIM with clinical characteristics to evaluate the risk of HT in patients who have undergone MT. This reliable model can assist frontline doctors in identifying patients with a significantly increased risk of HT and provide support during clinical decision-making.

## Data Availability

The raw data supporting the conclusions of this article will be made available by the authors, without undue reservation.

## Footnotes

^1^ http://www.itksnap.org/

^2^ http://pyradiomics.readthedocs.io

^3^ https://www.python.org
